# Characterization of the intestinal absorption of morroniside from *Cornus officinalis Sieb*. *et Zucc* via a Caco-2 cell monolayer model

**DOI:** 10.1371/journal.pone.0227844

**Published:** 2020-05-29

**Authors:** Renjie Xu, Hongdan Zhu, Lingmin Hu, Beimeng Yu, Xiaohua Zhan, Yichu Yuan, Ping Zhou

**Affiliations:** 1 Department of Clinical pharmacy, Shaoxing Women and Children’s Hospital, Shaoxing, Zhejiang, China; 2 Department of Pharmacy, Xinhua Hospital Affiliated to Shanghai Jiao Tong University School of Medicine, Shanghai, China; 3 Neonatal Intensive Care Unit, Shaoxing Women and Children’s Hospital, Shaoxing, Zhejiang, China; 4 Department of Laboratory, Shaoxing Seventh People’s Hospital, Shaoxing, Zhejiang, China; 5 The Third Maternal wards, Shaoxing Women and Children’s Hospital, Shaoxing, Zhejiang, China; 6 Department of Urology, The Second Affiliated Hospital, School of Medicine, Zhejiang University, Hangzhou, Zhejiang, China; University of Szeged, HUNGARY

## Abstract

Morroniside is a biologically active polyphenol found in *Cornus officinalis Sieb*. *et Zucc* (CO) that exhibits a broad spectrum of pharmacological activities, such as protecting nerves, and preventing diabetic liver damage and renal damage. However, little data are available regarding the mechanism of its intestinal absorption. Here, an *in vitro* human intestinal epithelial cell model of cultured Caco-2 cells was applied to study the absorption and transport of morroniside. The effects of donor concentration, pH and inhibitors were investigated. The bidirectional permeability of morroniside from the apical (AP) to the basolateral (BL) side and in the reverse direction was studied. When administered at three tested concentrations (5, 25 and 100 μM), the apparent permeability coefficient (P_app_) values in the AP-to-BL direction ranged from 1.59 × 10^−6^ to 2.66 × 10^−6^ cm/s. In the reverse direction, BL-to-AP, the value was ranged from 2.67 × 10^−6^ to 4.10 × 10^−6^ cm/s. The data indicated that morroniside transport was pH-dependent. The permeability of morroniside was affected by treatment with various inhibitors, such as multidrug resistance protein inhibitors MK571 and indomethacin, as well as the breast cancer resistance protein inhibitor apigenin. The mechanisms of the intestinal absorption of morroniside may involve multiple transport pathways, such as the passive diffusion and efflux protein-mediated active transport especially involving multidrug resistance protein 2 and breast cancer resistance protein. After the addition of CO, the P_app_ values in the AP-to-BL direction increased significantly, therefore, it can be assumed that some ingredients in the CO promote morroniside absorption in the small intestine.

## Introduction

**Fig 1:**
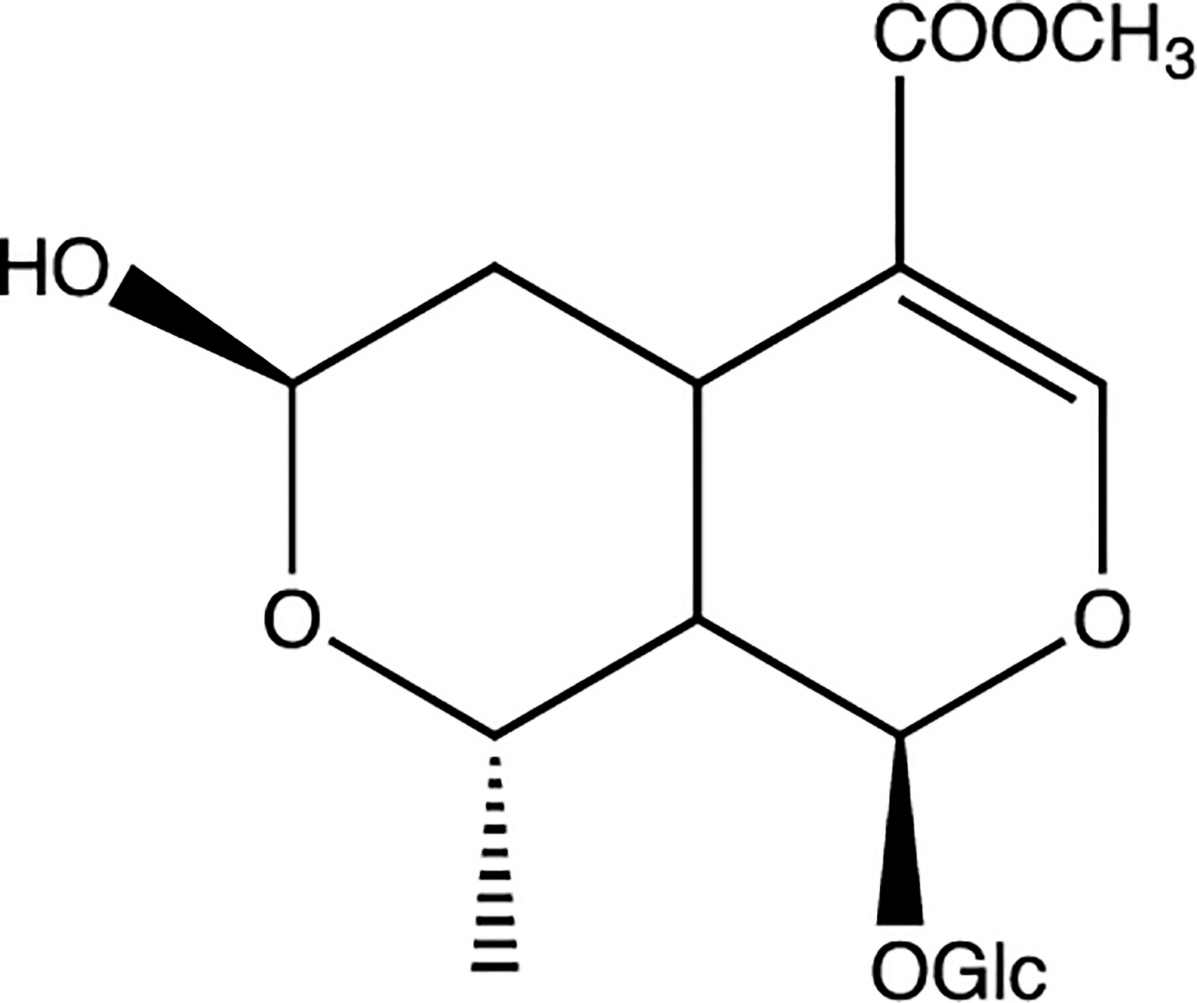
The chemical structure of morroniside.

Traditional Chinese medicines (TCMs) are natural therapeutic remedies that have been widely used for thousands of years [1]. Morroniside (Fig 1), one of the most important iridoid glycosides, is the main active ingredient of *Cornus officinalis Sieb*. *et Zucc* (CO). It is a rich source of iridoid glycosides and has been used as a traditional Chinese medicinal herb for centuries [2]. Various pharmacological studies have indicated that morroniside is effective in the treatment of Alzheimer’s disease [3], protecting nerves [4], preventing diabetic liver damage [5] and renal damage [6]. Morroniside also has beneficial effects on lipid metabolism and inflammation [7] and having anti-anaphylactic activity [8]. As morroniside and its correlative plant extracts exhibit pharmacological effects, it is hopeful that morroniside to be developed into promising preparations of herbal medicinal products.

Several studies have been conducted to determine the concentration of morroniside in biological matrices [9–11]. The absolute oral bioavailability of morroniside in rats was calculated to be only 3.6–7.0% [9, 12]. The plasma levels of morroniside administered intravenously are much higher than those after oral administration. It is well known that oral administration is the main route for the application of TCMs and that they should be absorbed in the gastrointestinal tract [13]. The intestinal absorption barrier is a major factor that controls the absorption and oral bioavailability of drugs [14–16] and the first steps of pharmacokinetics occur after oral intake. Therefore, exploration of the intestinal absorption mechanism of morroniside is necessary not only for an *in vivo* pharmacokinetics study but also to provide key information about its effective delivery system.

The aim of this present study was to investigate the intestinal absorptive characteristics of morroniside using Caco-2 cells. This model is extensively used because of its morphological and functional similarities to the human small intestinal epithelium, and it has been recognized by the Food and Drug Administration as a viable model that replicates human intestinal absorption [17–20]. The authors aimed to reveal the reason for the low bioavailability of morroniside and provide a theoretical basis for the development of formulations.

## Materials and methods

### Materials and reagents

Transwell permeable polycarbonate inserts (0.4 μm) and 12-well cell culture plates were obtained from Corning. (Cambridge, MA, USA). The Caco-2 cell line was generously provided by the Cell Bank of the Chinese Academy of Sciences (Shanghai, China). Dulbecco’s modified Eagle’s medium (DMEM) was obtained from Gibco Laboratories (Life Technologies Inc., Grand Island, NY, USA). Hanks’ balanced salt solution (HBSS, powder form) was obtained from Sigma Chemical Co. (Deisenhofen, Germany). Fetal bovine serum was purchased from HyClone (Logan, UT, USA). The 100× nonessential amino acids, 100× penicillin and streptomycin, 0.25% trypsin with ethylene-diaminetetraacetic acid (EDTA), 1 M 4-(2-hydroxyethyl)-1-piperazi- neethanesulfonic acid (HEPES), and bovine serum albumin were purchased from Invitrogen Corp. (Carlsbad, CA, USA). Verapamil, MK571, indomethacin, benzbromarone, apigenin, sodium vanadate and cimetidine used in this study were obtained from Aladdin Industrial Inc. (Shanghai, China). Morroniside (purity > 98.0%) and loganin (internal standard (IS), purity > 98.0%) were obtained from the National Institute for the Control of Pharmaceutical and Biological Products (Beijing, China). All other reagents (typically of analytical grade or better) were used as received.

### Herbal extract preparation

The extract was prepared according to a previously reported method [21]. The CO (100 g) crude material was decocted with 1000 mL of water for 2 h. Then, the filtrate was collected and the residue was decocted with 1000 mL water for another 2 h. Finally, the two batches of filtrates were combined and concentrated to 100 mL. The solution was centrifuged to pellet any insoluble molecules and then filter sterilized with a 0.22 μm proton exchange membrane (PEM) syringe filter. The content of morroniside in the preparation was approximately 16.02 mg/g in the crude material and 16.02 mg/mL in CO aqueous solution, as determined by high-performance liquid chromatography [9]. The pH of the preparation was approximately 5.5. In subsequent experiments, the CO solution was used for cytotoxicity and transport experiments. The final concentration of morroniside in the CO group in the next two experiments was approximately 25 μM.

### Caco-2 cell culture and cytotoxicity assay

The conditions for Caco-2 cell culture have been described previously [22]. Briefly, Caco-2 cells were cultured in a humidified atmosphere of 5% CO_2_ and 95% air at 37 °C. The culture medium (DMEM) was supplemented with 10% (v/v) fetal bovine serum, 1% nonessential amino acids, 1% penicillin, and streptomycin. The medium was changed every 2–3 days. When the cell monolayer reached 80–90% confluence, the cells were detached with a solution of trypsin (0.5 mg/mL) and EDTA (0.2 mg/mL) and reseeded at a density of 5×10^4^ cells/cm^2^.

For the cytotoxicity assay, the cells were incubated in 96-well plates for 24 h. Morroniside dissolved in methanol was diluted with DMEM to terminal concentrations that ranged from 0.1 μM to 200 μM, or CO at 0.63 mg/mL (containing 25 μM morroniside). A negative control group was obtained by the addition of DMEM with methanol at 0.5%(v/v). The cell culture medium treated group was treated only with DMEM. MTT solution was prepared to 1 mg/mL in phosphate-buffered saline and filtered through a 0.2 μm filter. Then, 20 μL of MTT was added into each well. Cells were incubated for 4 h at 37 °C with 5% CO_2_, 95% air and 100% humidity. After 4 h, the MTT solution was removed and replaced with 150 μL of DMSO. The plate was further incubated for 5 min at room temperature (20 °C), and the optical density of the wells was measured using a plate reader at a wavelength of 490 nm.

### Transport of morroniside across the Caco-2 monolayer

An initial stock solution of morroniside in methanol was prepared. The stock solution was then diluted with HBSS (pH = 7.4) and the final concentration of methanol in HBSS remained at 0.5%. The dissolution method of morroniside was described in a previous report [23], and residual methanol did not change the permeability results. Caco-2 cells were seeded at a density of 5 × 10^4^ cells/cm^2^ in a 12-well plate. The transepithelial electrical resistance (TEER) value was measured with a Millipore ERS voltameter (Burlington, MA, USA) in order to evaluate and determine monolayer integrity. The monolayers were ready for experiments 19 to 22 days after seeding. Only monolayers that demonstrated TEER values above 400 Ω × cm^2^ were used for the experiment.

During incubation, the culture medium was refreshed every other day. The culture medium was discarded, and 500 μL of HBSS was added to each well and incubated for 20 min at 37 °C. Afterward, each well was rinsed two times with HBSS at 37 °C. Three concentrations of morroniside (5, 25 and 100 μM) were added to either the apical (AP, 0.5 mL) or basolateral side (BL, 1.5 mL), wheras the receiving chamber contained the corresponding volume of blank HBSS medium (pH = 7.4). Incubation was performed at 37 °C for 180 min, with shaking at 50 rpm. To assess drug transport from the AP to BL side, after incubation for 5, 15, 30, 45, 60, 90, 120 or 180 min, 50 μL of the solution from the BL or AP side was collected, and replaced with an equal volume of HBSS. To study the effect of pH on morroniside (5, 25 and 100 μM) transport, the experiments were performed in HBSS, which was adjusted to pH 6.0 or pH 7.4 on the AP side depending on the experiment, and to pH 7.4 on the BL side at 37 °C.

Efflux and influx transporters were investigated for their effects on the transport flux of morroniside. Several ATP-binding cassette transporter inhibitors, including one p-glycoprotein (P-gp) inhibitor (100μM verapamil) [24]; one multidrug resistance- related protein (MRP) inhibitor (100 μM, MK571) [25], one multidrug resistance- related protein 3 (MRP3, the main BL MRP) inhibitor (50μM, benzbromarone) [26] and one multidrug resistance- related protein 2 (MRP2, the main AP MRP) inhibitor (200μM indomethacin) [27]; and one breast cancer resistance protein (BCRP) inhibitor (25μM apigenin) [28] were used to determine the transporters involved in the efflux transport of morroniside. The inhibition of the influx transport of morroniside transport across the Caco-2 cell monolayer was investigated by the addition of 50 μM sodium vanadate and cimetidine to evaluate the selectivity of Na/K-ATPase [23]. The pH and temperature conditions of this assay were 7.4 and 37 °C, respectively.

### Sample processing

Samples were treated using a protein precipitation method. After spiking with a 4-fold volume of acetonitrile that contained 5% acetic acid and 70 ng/mL IS as described in previous report [29], the tubes were vortexed for 1 min. The tubes were centrifuged at 14,000 rpm for 10 min, and the supernatants were collected and filtered through a 0.22 μm hydrophobic membrane [29]. An aliquot of 2 μL was injected into a liquid chromatography with tandem mass spectrometry (LC-MS/MS) system for analysis.

### LC-MS/MS analytical methods

An API 5500 triple-quadrupole mass spectrometer (Applied Biosystems-SCIEX, Concord, Canada) equipped with an electrospray ionization (ESI) source was used for chromatographic analyses. The separation was achieved with a ZORBAX Eclipse Plus C_18_ column (50 mm × 2.1 mm, 1.8 μm). The mobile phase consisted of 0.1% acetic acid in an aqueous solution as solvent A and 100% acetonitrile as solvent B, with a flow rate of 0.4 mL/min. The following gradient elution was used: 0–1.0 min 1% B; 1.0–2.2 min 1→29% B; 2.2–2.3 min 29→95% B; 2.3–3.3 min 95% B; 3.3–3.4 min 95–1% B, followed by a 1.0 min re-equilibration at the initial conditions. The column was maintained at 30 °C and the autosampler tray was also maintained at 30 °C.

Analytical procedures were evaluated with negative electrospray ionization (ESI) mode. Typical parameters for the Turbo ion spray source set were as follows: ion spray source temperature at 550 °C, ion spray voltage (IS) at 5000V; gas 1 and gas 2 (nitrogen) at 50 psi; CUR at 50; and CAD at -2.0. Other parameters were shown in Table 1.

**Table 1:** Optimized MRM parameters for the determination of morroniside and the IS.

Compound	Precursor ion, m/z	Product ion, m/z	DP (V)	CE (eV)	CXP (V)	Detected ion
**Morroniside**	451.0	243.0	-100	-30	-15	M^-^
**Loganin**	435.2	227.0	-100	-23	-15	M^-^

### Statistical analysis

UPLC-MS/MS data acquisition was performed using Analyst 1.5.2 and MultiQuant 2.1.1 software (Applied Biosystems). All data are shown as the mean ± standard deviation, n = 3. The data were analyzed using SPSS 20.0. In the Caco-2 cell model, the rate of transport was obtained from the amount transported versus the time curve using linear regression. The permeability coefficient of each compound was calculated using the following equation: P_app_ = (dQ/dt)/(A·C_0_).

where, dQ/dt is the rate of drug transport, C_0_ is the initial concentration of the compound in the donor chamber and A represents the surface area of the cell monolayer. The efflux ratio P was determined by calculating the ratio of P_app_ in the secretory (BA) direction divided by that in the absorptive (AB) direction, according to the following equation:

$$P=P_{app}BA/P_{app}AB$$

## Results

### Cytotoxicity in Caco-2 cells

**Fig 2:**
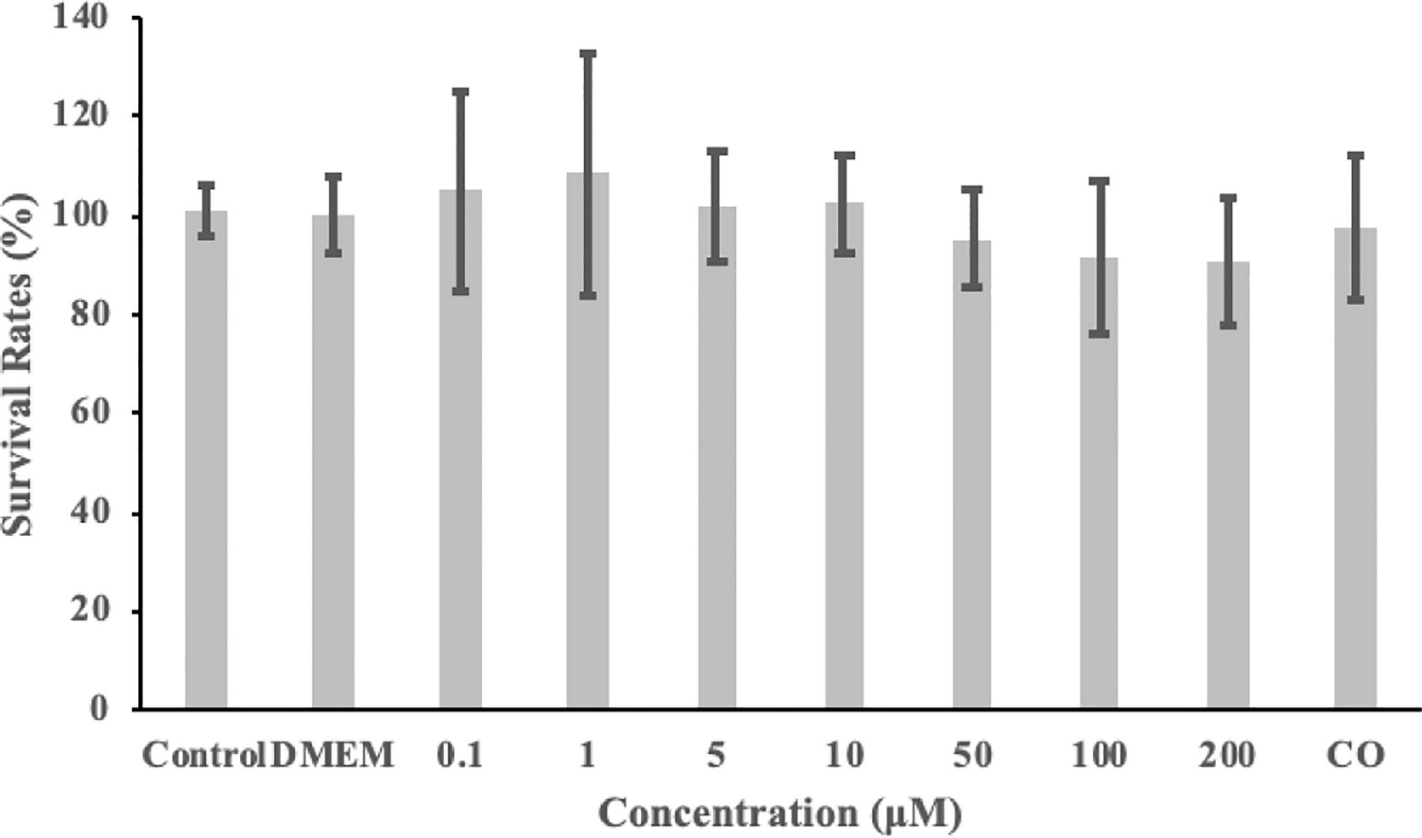
Cytotoxicity of morroniside or *Cornus officinalis Sieb*. *et Zucc* (CO) in Caco-2 cells as evaluated by the MTT assay. Data are the mean values ± standard deviation of five replicates.

The viability of the cells was directly measured using the MTT test to evaluate the cytotoxicity of morroniside toward Caco-2 cells prior to the transport experiments. As shown in Fig 2, morroniside could affect the viability of Caco-2 cells slightly although the difference was not statistically significant. Even at the highest concentration (200 μM), the viability of Caco-2 cells treated with morroniside was reduced by only 8%, which indicated that in our experimental design, morroniside was nontoxic to the growth of Caco-2 cells, as was 0.63 mg/mL CO (containing 25 μM morroniside).

### LC-MS/MS analysis to quantify morroniside

**Fig 3:**
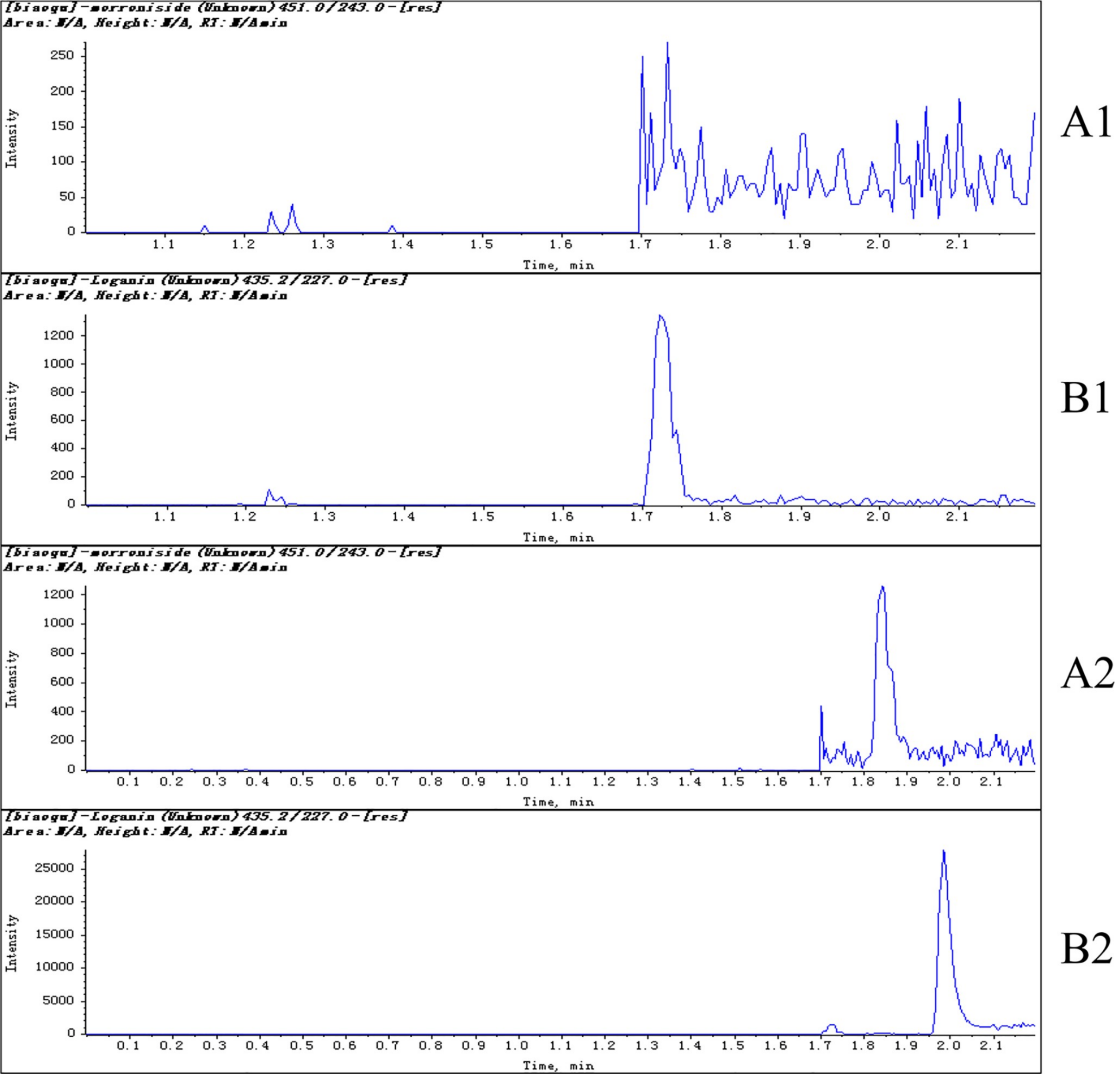
Representative multiple reaction monitoring chromatograms of morroniside [1] and IS [2] in fresh blank HBSS (A) and a sample (B) 5 min after the transport experiments by LC-MS/MS (7.81 ng/mL for morroniside and 70 ng/mL for IS).

The LC-MS/MS retention times for morroniside and the IS were approximately 1.85 and 2.01 min, respectively. Representative chromatograms are presented in Fig 3, including that of morroniside and IS in fresh blank HBSS and a sample 5 min after the transport experiments. No significant peaks interfering with morroniside or IS were observed in fresh blank HBSS (Fig 3). Calibration curves, constructed using linear least- squares regression, showed good linearity within the concentration range of 4–1000 ng/mL morroniside in HBSS. A typical calibration curve equation for morroniside was y = 0.007x+0.0055, where y represents the ratio of the morroniside peak area to the loganin peak area (and x is the concentration of morroniside). The calibration curves displayed good linearity as indicated by high correlation coefficients (R^2^ = 0.9997). The lower limit of quantification of the collected samples was 4 ng/mL.

### Transcellular transport of morroniside across the Caco-2 cell monolayers

The P_app_ values at the three concentrations of morroniside (5, 25 and 100 μM) in from either the AP to the BL side or in the reverse direction over 3 h are displayed in Table 2. As shown in Table 2, the P_app_AB of morroniside ranged from 1.59×10^−6^ cm/s at 100 μM to 2.66×10^-6^cm/s at 5 μM (AP to BL) and from 2.67×10^−6^ cm/s at 100 μM to 4.10×10^-6^cm/s at 5 μM (BL to AP). There was no significant difference in the P_app_ values at the three concentrations of morroniside (5, 25 and 100 μM) in form either the AP to the BL side or in the reverse direction.

**Table 2:** Transport of morroniside (5, 25 and 100 μM) across Caco-2 cell monolayers (n = 3).

Concentration (μM)	P_app_AB_(×10_^-6^_cm/s)_	
	AP-BL	BL-AP
5	2.66±0.60	4.10±0.86
25	1.83±0.65	2.92±0.63
100	1.59±0.41	2.67±0.48

### Effect of pH on the permeation of morroniside

The assay of AP to BL transport in HBSS at two pH values is shown in Table 3. The P_app_AB values of morroniside at the three concentrations at pH 7.4 were significantly higher than those at pH 6.0 (p<0.05) (Table 3), which indicated an easier transport of morroniside at pH (7.4) than at pH (6.0).

**Table 3:** P_app_AB of morroniside (5, 25 and 100 μM) in human intestinal Caco-2 cells treated at different pH values at 37 °C (n = 3, p < 0.05).

Conditions	P_app_AB_(×10_^-6^_cm/s)_		
	5μM	25μM	100μM
pH 7.4	1.88±0.49	1.92±0.52	1.86±0.33
pH 6.0	0.55±0.07*	0.61±0.15*	0.62±0.10*

* Denotes results that are significantly different from those of the pH 7.4 experiments (p < 0.05).

### Effects of various compounds on morroniside transport

With the participation of 50 μM sodium vanadate or cimetidine, the transport of morroniside did not significantly increase or decrease. These results indicated that influx transporters such as OATs or Na^+^/K^+^ pumps contributed little to the transport of morroniside (Table 4).

**Table 4:** Inhibitory effects of influx transporters on morroniside transport in Caco-2 cell monolayers (n = 3).

Inhibitor	Transporter	P_app_AB_(×10_^-6^_cm/s)_	P_app_BA_(×10_^-6^_cm/s)_	P
**Control**		1.90±0.5	3.06±0.6	1.60
**Sodium vanadate**	Na^+^/K^+^pump	1.89±0.4	3.11±0.7	1.65
**Cimetidine**	OATs	1.82±0.5	3.1±0.7	1.70

**Fig 4:**
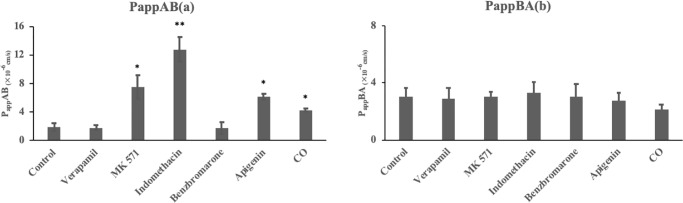
Inhibitory effects of efflux transporters on morroniside (25 μM) transport in human intestinal Caco-2 cell monolayers: AP to BL (a) and from BL to AP (b) (n = 3). *Denotes results that are significantly different from those of the control experiments (p < 0.05). **Denotes results that are significantly different from those of the control experiments (p < 0.001).

The inhibitory effects of efflux transporters on morroniside transport in Caco-2 cell monolayers are shown in Fig 4 with the corresponding efflux ratio P values in Table 5. To determine whether P-gp is involved in the transport of morroniside, its transport was studied in the presence of verapamil, a known inhibitor of P-gp. However, the P_app_AB and P_app_BA of morroniside did not change significantly before and after the addition of verapamil, which indicated that P-gp might not be involved in the transport of morroniside (Fig 4).

**Table 5:** Efflux ratio of efflux transporters involved in morroniside transport in Caco-2 cell monolayers.

Efflux ratio	Control	Verapamil	MK 571	Indomethacin	Benzbromarone	Apigenin	CO
P	1.61	1.59	0.40	0.26	1.72	0.45	0.49

MRP and BCRP are present in the intestinal tract of humans, and are responsible for the transport and efflux of compounds and drugs [30]. Fig 4 shows that the P_app_AB values increased after MK 571 was added, but they had no effect on P_app_BA. The data indicated that morroniside was effluxed by MRP. MRP has different isoforms, of which MRP2 is located at AP side in the cell membrane and MRP3 is localized on the BL side [31]. The results in Fig 4 show that the P_app_AB values caused a significant increase in the AP to BL flux (p < 0.001) after indomethacin was added. This in turn indicated that morroniside is a substrate of MRP2. Cotreatment with 200 μM benzbromarone did not alter the P_app_AB or P_app_BA values of morroniside, and the efflux ratio was reduced by only 6.83%.

BCRP is located on the AP side of the cell membrane [32]. Apigenin was used to determine the effects of BCRP on the transport of 25 μM morroniside. After the addition of apigenin (25 μM), the P_app_AB of morroniside increased significantly (Fig 4, p < 0.05), which fresulted in a decrease in the efflux ratio by 72.05% (Table 5). Therefore, it can be assumed that morroniside is a substrate of BCRP.

## Discussion

The cytotoxicity assay, showed that concentrations of morroniside between 0.1 and 200 μM could be used for the subsequent transport experiments. We referred to the concentration selections of some structural analogs of morroniside such as geniposide [33], gentiopicroside [34] and loganin [35] from other articles and 5, 25 and 100 μM were selected.

As shown in Table 2, there was no significant difference among the three concentration groups. However, it still seemed that P_app_ increased as the concentration of morroniside decreased. This phenomenon is somewhat similar to that in other compounds such as epimedin C [36]. The apparent permeability coefficient decreased with increasing concentrations over the experimental range. The behavior in both directions may be consistent with a saturable mechanism according to some previous reports [31, 36]. Additional concentrations and replicates are still needed to explore the relationship between the concentration of morroniside and P_app_ values.

To further study the transport characteristics of morroniside, we investigated the relationship between time and permeated amount to transport. The permeated amount of morroniside increased approximately linearly with time from 0 to 60 min. After 60 min, the permeated amount gradient between the two sides greatly decreased, which resulted in the curves reaching a plateau (S1 Fig). A possible reason for this is that the concentration gradient between the two sides greatly decreased after 60 min, which resulted in reduced transport that led to the plateau. Therefore, it was concluded that passive transfer is involved in the intestinal absorption mechanism of morroniside. In our previous work, loganin [35], a structural analog of morroniside, showed good intestinal permeability using the human intestinal Caco-2 cell model. It seemed that the cell permeability of morroniside was lower than that of loganin.

Accounting for the limit of detection of the method, compounds that are completely absorbed in the human intestine typically exhibit P_app_ values of >70×10^−6^ cm/s in the Caco-2 transwell system, whereas compounds with poor absorption (<20%) have P_app_ values of <10×10^−6^ cm/s [37]. The determined P_app_ values of morroniside were at the level of 10^−6^ which indicated poor intestinal absorption of morroniside. It is also important to note that, strong metabolism may be regarded as another major cause of the low oral bioavailability of morroniside *in vivo*. However, further studies are required to clarify whether metabolism is the main factor in the *in vivo* processing of morroniside.

The P_app_BA values of morroniside were higher than the P_app_AB values (p < 0.05), which indicated that some transporters might be involved in the transport of morroniside in the BL to AP direction. (Table 2).

The data illustrating how pH affects the permeation of morroniside, show that morroniside transport is pH-dependent. These results indicate that transporters may be involved in the efflux of morroniside. In fact, previous research has indicated that both the influx [38] and efflux transport such as BCRP [39, 40] are activated at lower pH levels. Therefore, it can be assumed that morroniside is also a substrate of BCRP. This can be verified in subsequent experiments.

The efflux protein MRP2 is localized on the AP side. The transport of morroniside from the AP to the BL side could be inhibited by MRP2. Indomethacin is a MRP2 inhibitor. The efflux effect of MRP2 was reduced after indomethacin addition, which resulted in the higher permeability of morroniside from the AP to the BL side than in the control group.

The three experiments, which contained the pH experiment, concentration experiment and inhibitor experiment, were independent. The control groups in the experiments were not significantly different.

After 0.63 mg/mL CO (containing 25 μM morroniside) was added, the P_app_AB increased significantly, and the P_app_BA value was not significantly different from that the control group. After the CO preparation was added to the AP or BL side with HBSS in the transport experiment, the pH was approximately 7.4. The small amount of CO preparation did not change the final pH during the transport experiment. Many ingredients, especially iridoid glycosides, are present in CO. To our knowledge, the most abundant bioactive iridoid glycosides, which are widely distributed in CO, are loganin, morroniside and sweroside [41]. Loganin, which was discovered to be a substrate of BCRP in our previous study may reduce the efflux of morroniside and lead to a higher P_app_AB in the crude material group [35]. However, the compounds contained in CO are complex and further research is needed to clarify the intestinal absorption characteristics of this herbal preparation.

## Conclusions

In the present study, morroniside was shown to be a poorly absorbed compound in the Caco-2 cell monolayer model, and the mechanisms were passive diffusion and efflux protein-mediated active transport. The transporters, BCRP and MRPs (especially MRP2) are vital for morroniside transport in the intestine. These novel results provide useful information for the prediction of the oral bioavailability, pharmacokinetics, and clinical applications of morroniside as well as for the determination of the substance basis for its bioactivity.

## Supporting information

S1 Fig Time-course of morroniside (5, 25 and 100μM) transport across the Caco-2 cell monolayers from apical (AP) to basolateral (BL) and from BL to AP (n = 3). (DOCX)

S1 Dataset Minimal data set. (DOCX)
