# Supplementary material for: Characterization of the intestinal absorption of morroniside from Cornus officinalis Sieb. et Zucc via a Caco-2 cell monolayer model
Source: PLoS One. 2020 May 29;15(5):e0227844. doi: 10.1371/journal.pone.0227844 (PMC7259638; doi:10.1371/journal.pone.0227844)
Supplement: S1 Fig — (DOCX) [file pone.0227844.s001.docx]

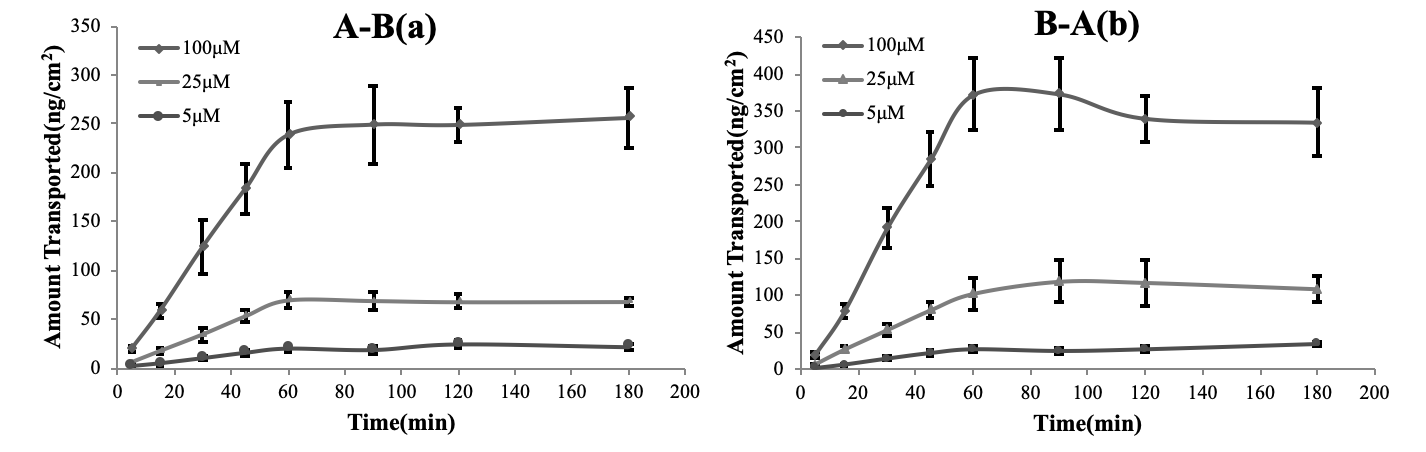


S1 Fig. Time-course of morroniside (5, 25 and 100μM) transport across the Caco-2 cell monolayers from AP to BL and from BL to AP (n=3)
