# Supplementary material for: Characterization of the intestinal absorption of morroniside from Cornus officinalis Sieb. et Zucc via a Caco-2 cell monolayer model
Source: PLoS One. 2020 May 29;15(5):e0227844. doi: 10.1371/journal.pone.0227844 (PMC7259638; doi:10.1371/journal.pone.0227844)
Supplement: S1 Dataset — (DOCX) [file pone.0227844.s002.docx]

**Minimal data set**

| Concentration (μM) | Survival Rates (%, mean) | SD |
| --- | --- | --- |
| **Control** | 101.12 | 5.23 |
| **DMEM** | 100 | 7.99 |
| **0.1** | 105.12 | 20.11 |
| **1** | 108.33 | 24.33 |
| **5** | 101.62 | 10.93 |
| **10** | 102.41 | 10.05 |
| **50** | 95.21 | 10.12 |
| **100** | 91.77 | 15.34 |
| **200** | 90.72 | 12.88 |
| **CO** | 97.33 | 14.55 |

Fig 2 Cytotoxicity of morroniside or *Cornus officinalis Sieb. et Zucc* (CO) in Caco-2 cells as evaluated by the MTT assay. Data are the mean values ± standard deviation of five replicates.

| Inhibitors | Control | Verapamil | MK 571 | Indomethacin | Benzbromarone | Apigenin | CO |
| --- | --- | --- | --- | --- | --- | --- | --- |
| P_app_AB (mean) | 1.9 | 1.84 | 7.55 | 12.8 | 1.77 | 6.12 | 4.284 |
| P_app_AB (SD) | 0.5 | 0.3 | 1.6 | 1.7 | 0.81 | 0.4 | 0.2 |
| P_app_BA (mean) | 3.06 | 2.93 | 3.01 | 3.3 | 3.04 | 2.78 | 2.112 |
| P_app_BA (SD) | 0.6 | 0.7 | 0.4 | 0.8 | 0.9 | 0.5 | 0.4 |

Fig 4 Inhibitory effects of efﬂux transporters on morroniside (25 μM) transport in human intestinal Caco-2 cell monolayers: AP to BL (a) and from BL to AP (b) (n = 3).

*Denotes results that are signiﬁcantly different from those of the control experiments (p < 0.05).

**Denotes results that are signiﬁcantly different from those of the control experiments (p < 0.001)
